# Green tea extract promotes DNA repair in a yeast model

**DOI:** 10.1038/s41598-019-39082-9

**Published:** 2019-03-07

**Authors:** Shin Yen Chong, Hsin-Yi Chiang, Tzu-Hung Chen, Yi-Ju Liang, Yi-Chen Lo

**Affiliations:** Graduate Institute of Food Science and Technology, National Taiwan University, No. 1, Sec. 4, Roosevelt Rd., Da’ an Dist., Taipei 10617 Taiwan

## Abstract

Green tea polyphenols may protect cells from UV damage through antioxidant activities and by stimulating the removal of damaged or cross-linked DNA. Recently, DNA repair pathways have been predicted as possible targets of epigallocatechin gallate (EGCG)-initiated signaling. However, whether and how green tea polyphenols can promote nucleotide excision repair and homologous recombination in diverse organisms requires further investigation. In this report, we used the budding yeast, *Saccharomyces cerevisiae*, as a model to investigate the effects of green tea extract on DNA repair pathways. We first showed that green tea extract increased the survival rate and decreased the frequency of mutations in yeast exposed to UVB-irradiation. Furthermore, green tea extract increased the expression of homologous recombination genes, *RFA1*, *RAD51* and *RAD52*, and nucleotide excision repair genes, *RAD4* and *RAD14*. Importantly, we further used a specific strand invasion assay to show that green tea extract promotes homologous recombination at double-strand breaks. Thus, green tea extract acts to preserve genome stability by activating DNA repair pathways in yeast. Because homologous recombination repair is highly conserved in yeast and humans, this study demonstrates yeast may be a useful platform for future research to investigate the underlying mechanisms of the bioactive compounds in DNA repair.

## Introduction

Over the past decades, many studies have demonstrated that green tea and its bioactive components are beneficial to human health. Most of the beneficial effects of green tea have been attributed to the robust anti-inflammatory and anti-tumorigenic effects of constituent polyphenolic catechins, such as (–)-epigallocatechin-3-gallate (EGCG), (–)-epigallocatechin (EGC), (–)-epicatechin-3-gallate (ECG) and (–)-epicatechin (EC)^1–4^. While these physiological effects are well documented, the mechanisms by which catechins may protect from tumorigenesis are not fully described.

Genomic instability leads to mutations and promotes cancer formation in complex organisms^5^. Moreover, maintenance of genome stability is critical for normal cell growth, and prevents damaged or mutated DNA from being inherited by the next generation in all organisms. In order to maintain genome stability, highly conserved DNA repair systems were evolved in ancient ancestors to minimize the cytotoxic and mutagenic effects of DNA damage. One of the most common DNA damaging agents, which can lead to mutagenesis and skin cancer in humans, is UV-irradiation from sunlight^6^. UV-irradiation causes multiple types of DNA damage, including oxidative damage^7^, cross-linking of bases^8^ and double-strand breaks (DSBs), which are most detrimental to genome integrity^9^. Accumulation of UV-induced DNA lesions undoubtedly increases the probability of cancer formation. Indeed, an early study has demonstrated that the incidence of UV-induced skin cancer in mice can be reduced by enhancing DNA repair by applying exogenous T4 endonuclease V, which can initiate the removal of UV-induced cyclobutane pyrimidine dimers (CPDs) from DNA^10^.

A number of phenolic compounds, including vanillin, cinnamaldehyde, coumarin and tannic acid, prevent mutagenesis in *E*. *coli*, by influencing DNA replication and/or repair after DNA damage^11–13^. Several studies have shown that green tea extract (GTE) contains polyphenols that may act as antioxidants to prevent DNA damage^14–16^. A recent study revealed that regular intake of green tea can lower lymphocytic DNA damage and increase the activity of 8-oxoguanine glycosylase (OGG1), a DNA glycosylase enzyme involved in base excision repair (BER) in human lymphocyte extracts^17^. Moreover, green tea polyphenols were also shown to enhance CPD removal in skin cells by nucleotide excision repair (NER) and decrease apoptosis in mice after UV exposure^18–20^. Interestingly, EGCG has been shown to prevent cell cycle progression in cancer cells^21,22^ and decrease UVB-induced oxidative stress in human skin cells^23^. Intriguingly, homologous recombination (HR) has been recently predicted as a possible target of EGCG in a bioinformatics study on breast cancer^24^. However, it is unknown whether green tea polyphenols can promote NER and HR in diverse model organisms that may be useful for further mechanistic studies.

Yeast has been extensively used as an experimental organism for modern biology, especially due to its amenability to classical and molecular genetic methods. This organism is often used to associate genes with certain functions in eukaryotic cells, as it is genetically tractable and many biological mechanisms are highly conserved in humans^25^. For example, yeast models have been invaluable in dissecting the mechanisms by which phytochemicals in food (e.g. polyphenols from apples and tangeretin from orange peels) provide well-known benefits to human health^26,27^. In this study, we utilized the budding yeast *Saccharomyces cerevisiae* to investigate whether GTE could promote genome stability by regulating NER and HR, as most DNA repair pathways are highly conserved between yeast and humans^28^.

## Results

### Polyphenol content in green tea extract

We first determined the content of various compounds in GTE by HPLC. The concentrations of major catechins, including EGCG, EGC, ECG and EC, as well as gallic acid and caffeine in GTE (μg/100 ml) are shown in Table 1. The total catechin content in our GTE (~121 mg/100 ml) was comparable to those reported in a survey of nine green teas (74–216 mg/100 ml)^14^. Interestingly, our GTE had relatively low EGCG content, but much a higher level of EC. These differences with previous literature may be due to variations in production conditions and technologies^14^.

**Table 1:** Caffeine, catechin and gallic acid content in green tea extract, as measured by HPLC.

Component	(μg/100 ml GTE)
Caffeine	19046 ± 147^*a*^
EC^*b*^	5774 ± 174
ECG^*c*^	2008 ± 19
EGC^*d*^	30266 ± 671
EGCG^*e*^	12884 ± 649
Gallic acid	176 ± 6

^*a*^Values are mean ± S.D. from triplicate analysis.

^*b*^EC: epicatechin; ^*c*^ECG: epicatechin gallate; ^*d*^EGC: epigallocatechin;

^*e*^EGCG: epigallocatechin gallate.

### GTE treatment enhances the survival rate of UVB-irradiated cells

**Figure 1:**
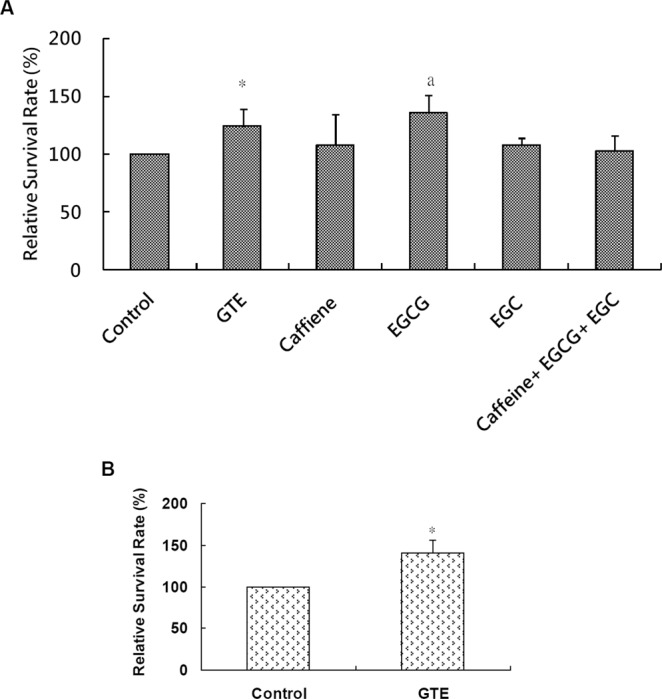
GTE-treatment enhances the survival of UVB-irradiated cells. Cells were irradiated with UVB at doses of (**A**) 200 J/m^2^ and (**B**) 400 J/m^2^. Relative percentage of surviving cells was calculated in comparison to UVB-irradiated controls. Data are presented as mean ± S.D. determined from at least two independent cultures measured in triplicate. *P < 0.05 compared to control. ^a^*P* = 0.05 compared to control.

We examined how GTE and its major polyphenolic components, EGCG, EGC and caffeine, affect cell survival after UVB-irradiation. GTE- and EGCG-treated groups showed significant increases (*P* < 0.05 and *P* = 0.05, respectively) in cell survival rates, as compared to untreated and other groups at a dose of 200 J/m^2^ UVB (Fig. 1A). In contrast, the survival rates of cells treated with EGC, caffeine, or a combination of EGCG, EGC and caffeine did not differ from the control (Fig. 1A). To test whether GTE could enhance survival of UVB-irradiated cells at higher doses, cells were exposed to a UVB dose of 400 J/m^2^, and the survival rates were determined. Indeed, we observed a significantly higher (*P* < 0.05) survival rate in the GTE-treated group compared to control (Fig. 1B). Thus, cells treated with GTE were more tolerant to UV-damage as compared to untreated cells or those treated with certain pure compounds.

### GTE promotes expression of NER genes

**Figure 2:**
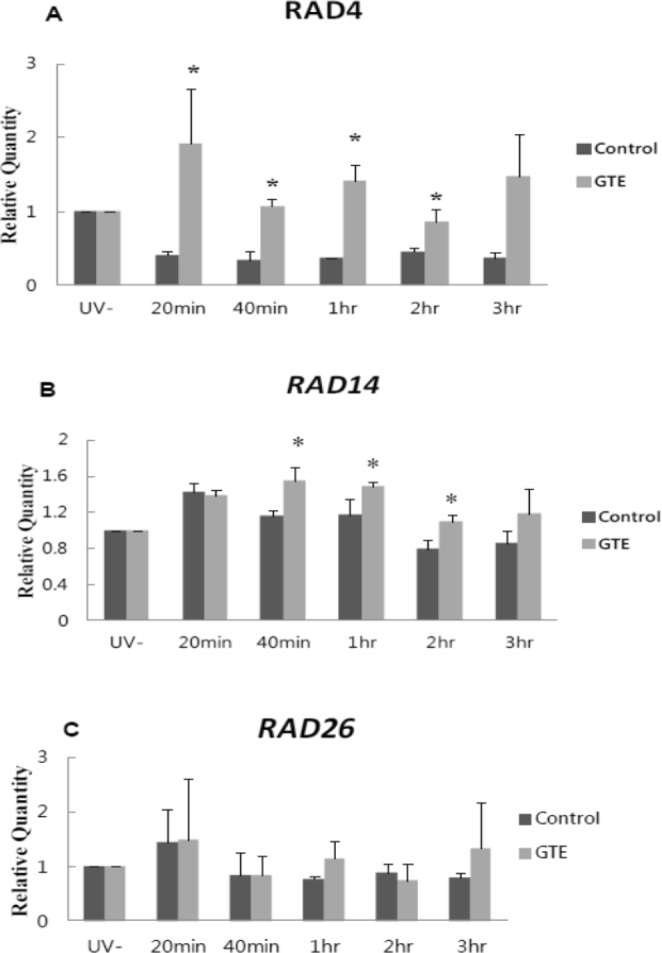
GTE treatment enhances expression of GG-NER genes following UVB irradiation. Expression levels of (**A**) *RAD4*, (**B**) *RAD14* and (**C**) *RAD26* are shown. Samples were collected before and after UVB-irradiation (200 J/m^2^) at the indicated time-points. White bars represent control groups and gray bars represent GTE groups. Data are presented as means ± S.D. *P < 0.05 compared to control at the same time-point, according to Student’s *t*-test.

Green tea polyphenols have been shown to enhance the removal of highly mutagenic UV-induced adducts from DNA^17–19^, a process that is predominantly mediated by NER. The NER pathway is divided into two sub-pathways: global genomic NER (GG-NER), which repairs transcriptionally inactive or silent areas of the genome, and transcription-coupled NER (TC-NER), which repairs DNA lesions in the coding regions of active genes^29^. To understand which sub-pathway is influenced by GTE, we investigated the expression of genes involved in both NER sub-pathways. We first examined expression of *RAD4* and *RAD14*, which encode key components of the GG-NER pathway^30^. *RAD4* (the yeast homolog of human xeroderma pigmentosum C: *XPC*) encodes a DNA damage-binding protein that plays key roles in the early steps of GG-NER^30^. The product of *RAD14* (a homolog of human xeroderma pigmentosum A: *XPA*) recognizes and binds to damaged DNA in both GG-NER and TC-NER^31^. GTE significantly enhanced (*P* < 0.05) expression of *RAD4* from 20 min to 2 h, and *RAD14* from 40 min to 2 h post-irradiation, as compared to the untreated group (Fig. 2A,B). However, GTE did not significantly affect expression of *RAD26* (the yeast homolog of human Cockayne Syndrome B: *CSB*), which encodes an essential component of the TC-NER pathway (Fig. 2C). These results suggest that GTE may specifically upregulate the GG-NER repair pathway by activating genes encoding DNA-damage-sensing Rad14 and DNA-binding Rad4.

### GTE promotes HR repair

**Figure 3:**
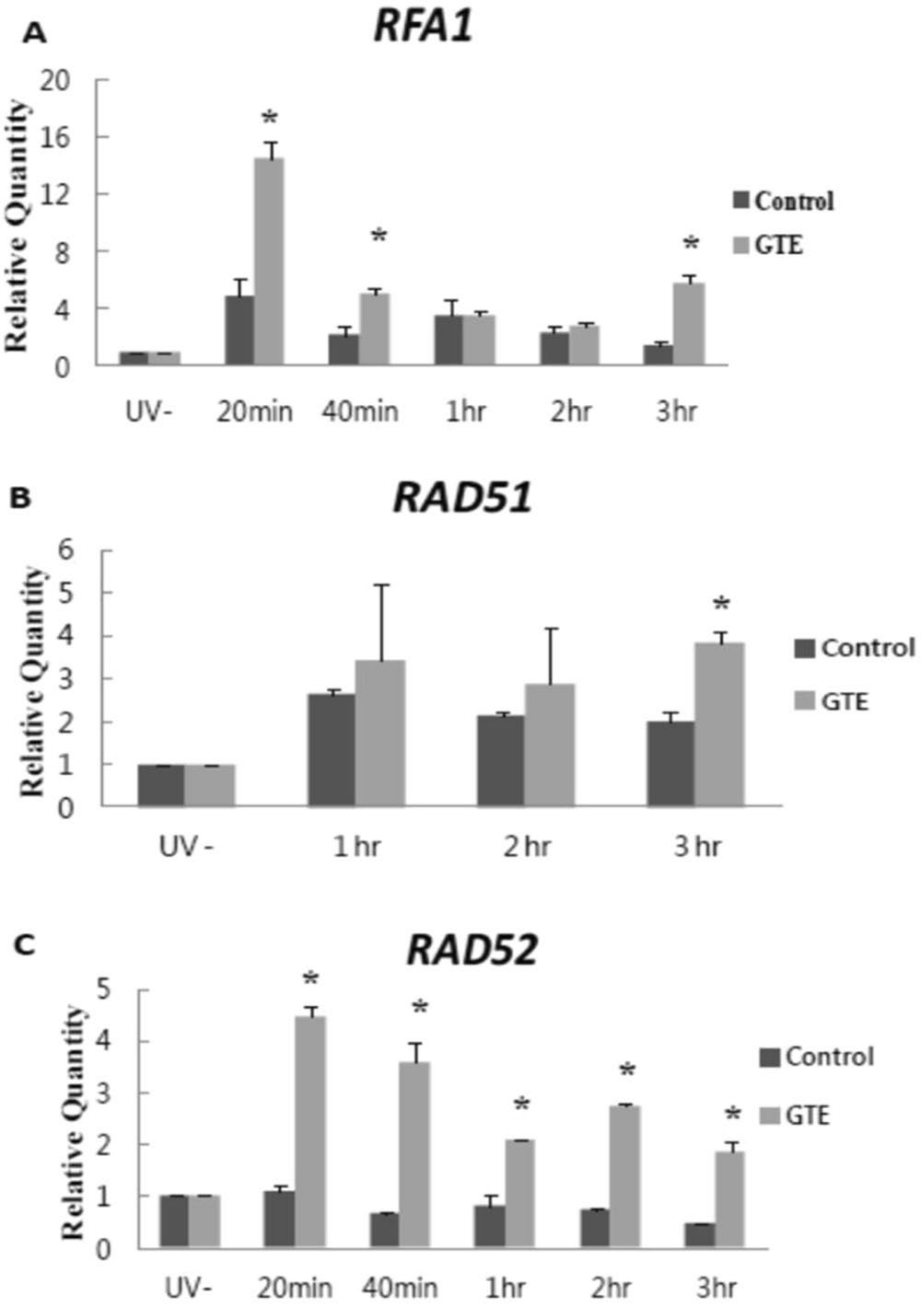
GTE treatment increases expression of HR genes following UVB irradiation. Expression levels of (**A**) *RFA1*, (**B**) *RAD51* and (**C**) *RAD52* are shown. Samples were collected before and after UVB-irradiation (200 J/m^2^) at the indicated time-points. Data are presented as means ± S.D. *P < 0.05 compared to control at the same time-point, according to Student’s *t*-test.

In addition to NER, HR is also involved in the repair of UV-induced DNA damage in yeast and human cells^32,33^, and its inactivation compromises yeast survival following UV-irradiation^33^. Thus, we further explored whether genes involved in the early stage of HR, including *RFA1*, *RAD51* and *RAD52*, were also activated by GTE. We found that *RFA1* expression was increased 20 min post-irradiation in GTE-treated cells, and expression was also elevated 3 h post-exposure (Fig. 3A). *RAD51*, which encodes a recombinase, also showed significant induction (*P* < 0.05) in cells treated with GTE at 3 h post-irradiation (Fig. 3B). Moreover, *RAD52* expression levels were significantly elevated (*P* < 0.05) in GTE-treated cells at all times examined following exposure to UVB (Fig. 3C). These results suggest that the HR pathway is upregulated by GTE treatment in response to UV damage.

**Figure 4:**
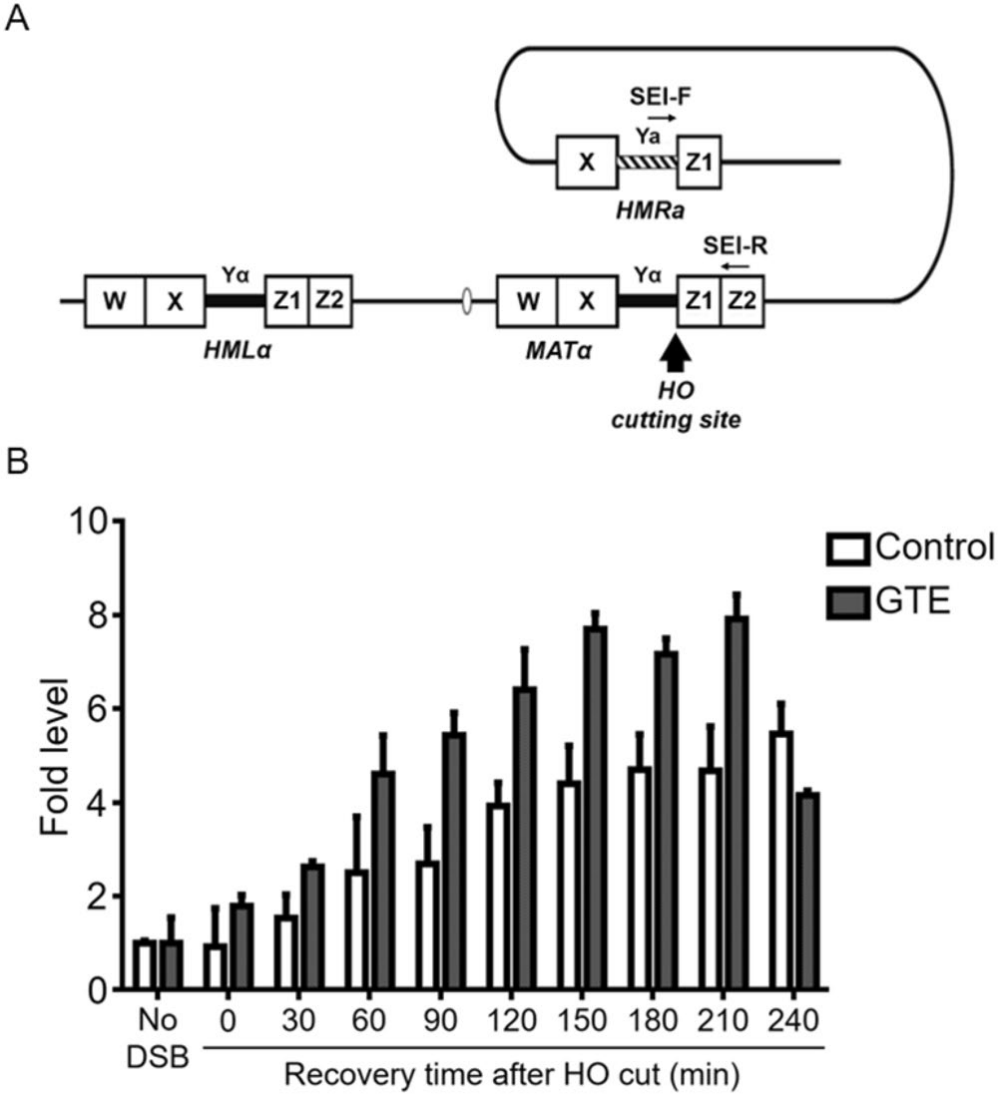
GTE promotes the HR activity. (**A**) Schematic of the *MAT* locus, *HML*, and *HMR* used for the single-end invasion (SEI) assay. The *MAT* locus, *HML*, and *HMR* share identical W, X and Z1 sequences. Galactose-induction of *pGAL*-HO endonuclease initiates the formation of a double-strand break at the *HO* cutting site. Repair of the double-strand break may occur via strand invasion by the *HMRa* region, which can be detected using qPCR with primers for SEI-F and SEI-R. (**B**) GTE enhanced HR repair at DSB sites, as measured by the SEI assay. White bars represent the control group and gray bars represent the GTE-treated group. Data are presented as means ± S.D.

To assess whether GTE is able to enhance HR activity, we used a strand invasion/repair (SEI) assay (Fig. 4A). In this assay, a specific DSB can be induced at the *MAT* locus through the induction of an exogenous site-specific HO endonuclease. To determine whether GTE altered the efficiency of DSB repair by HR, we measured the initial strand-invasion phase using the SEI assay. This PCR-based assay allows us to measure the intermediates of the strand invasion/repair synthesis reaction (Fig. 4A). Our results show that at 60 min after DSB induction, SEI was dramatically enhanced in GTE-treated cells as compared to control cells (Fig. 4B). SEI peaked at 210 min in GTE-treated cells and declined thereafter, probably indicating the completion of HR repair. In control cells, SEI continued to increase slowly throughout the experimental duration. These results suggest that GTE enhances the rate of initial strand invasion and repair synthesis phase during HR, likely by inducing the expression of HR repair genes.

### UVB-induced *CAN1* gene mutations suppressed by GTE

To investigate whether GTE-mediated induction of DNA repair genes results in suppression of UVB-induced gene mutations, we monitored the mutation rate of the *CAN1* gene. We found that irradiation with 200 J/m^2^ UVB increased the frequency of Can^*r*^ cells by about 40-fold in wild-type cells (Table 2). While treatment with GTE did not affect the spontaneous mutation rates, it markedly reduced the mutation frequency in irradiated cells, as compared to the irradiated control. GTE-treated cells exhibited a significantly lower *CAN1* gene mutation rate upon irradiation (28-fold over non-irradiated), as compared to cells treated with pure compounds (36-fold for EGCG; 35-fold for caffeine; Table 2). These results suggest that total GTE is more potent for protecting and maintaining genome stability after UV exposure than any single functional compound that we tested. Finally, GTE-treatment did not reduce the gene mutation rate in HR-defective *rad52∆* mutants (Table 2), an observation which is consistent with the notion that the protective effect of GTE is primarily through modulation of HR gene expression. Importantly, however, these results demonstrate that UVB-induced *CAN1* gene mutations may be suppressed by GTE.

**Table 2:** *CAN1* gene mutation rates.

Strains	Groups	Mutation rate/10^7^ cells	Fold increase
Wild type	Control	1.48	1
	Control + UV	57.61	38.95
Wild type	GTE	2.55	1.72
	GTE + UV	41.04*	27.74
Wild type	EGCG	1.15	0.78
	EGCG + UV	53.32	36.04
Wild type	Caffeine	1.09	0.74
	Caffeine + UV	52.17	35.27
*rad52Δ*	GTE	12.50	8.45
	GTE + UV	67.30	45.47

Data are presented as the median of five to ten independent colonies.

Values marked with * are significantly less than Control + UV, *P* < 0.05 by Mann-Whitney U test.

## Discussion

In the present study, we found that GTE enhances expression of DNA repair genes in response to UVB exposure. Interestingly, GTE was specifically found to enhance HR activity in order to protect against UV-induced gene mutations. Such effects on gene expression are likely to contribute to the enhanced survival observed in GTE-treated cells.

DNA repair plays an essential role in protecting the genome from endogenous and exogenous damage. When DNA is damaged, it must be rapidly and efficiently repaired. In humans, the XPC protein forms a damage recognition complex with HR23B to detect UV-induced DNA lesions^34,35^. XPA subsequently interacts with replication protein A (RPA) to bind and remove damaged DNA. Thus, cells that carry defective XPC and XPA are extremely sensitive to UV, and have very low NER activity^36^. Patients with mutations in these genes are predisposed to skin cancer and other systemic conditions. We have demonstrated that GTE activates expression of the *RAD4* and *RAD14* genes within minutes of UV exposure in wild-type yeast. Given that Rad4 and Rad14 are responsible for recognizing UV-damaged nucleotides in the indiscriminate genome-wide NER repair pathway, activation of these two genes may help to maintain the overall stability of the genome. Thus, our findings identify GTE as a possible supplement to enhance GG-NER to combat UV-initiated damage.

HR repair at the *MAT* locus in yeast is highly dependent on Rad52 and Rad51 proteins, and has been used extensively to assess HR activity. The *RFA1*, *RAD51* and *RAD52* genes are required for UV-induced HR repair in both yeast and human cells^37,38^. We demonstrated that GTE stimulates expression of *RFA1*, *RAD51* and *RAD52*, and this increased expression may translate into enhanced HR-mediated repair of DSBs (Fig. 4B). UV-irradiation may result in a single-strand break, and pairing of the exposed single-stranded DNA with homologous DNA allows HR repair to occur^39^. Consistent with this idea, we observed that a key HR gene, *RAD51*, was induced after (60 min after UVB treatment, Fig. 4B) the time at which we observed activation of NER genes (20 min after UVB treatment, Fig. 2A,B); this upregulated expression of *RAD51* then persisted for more than 3 h. Importantly, we observed that HR repair required nearly 4 h to complete according to our SEI assay (Fig. 4). Thus, GTE may promote HR repair when DSBs are encountered. Together, our results suggest that GTE treatment can sequentially enhance NER and HR DNA repair to allow cells to recover from UV-induced DNA damage.

Previous reports have argued that mutagenesis is induced shortly after irradiation, due to faulty repair or lack of repair before or during DNA replication^40^. Our data suggest that GTE positively regulates the expression of repair genes for at least 3 h (Figs 3 and 4), and long-term treatment with GTE suppresses UVB-induced mutagenesis (Table 2). It is well documented that delayed mutations can arise many cell generations after UV damage, thereby increasing the gene mutation rate in the genome^40–44^. Thus, our findings indicate that continuous application of GTE to the cells decreases the incidence of delayed mutations, contributing to improved survival and genome stability in the cells.

A previous study in yeast showed that cooperative action of all apple components has more anti-aging power than individual components^27^. Similarly, while EGCG has been suggested to be the major bio-effective factor in GTE, we observed that GTE was more effective than EGCG at promoting cell survival and reducing gene mutation rate. This improved effect may be due to the existence of other components in GTE, such as chlorophylls and pheophytin, which also function as antioxidants, anti-genotoxic and tumor-suppressing agents^44–47^. Thus, our results suggest that maintenance of genome stability after UV-damage by GTE may not be solely an effect of EGCG; instead, a combination of bioactive compounds in GTE may function together to suppress UVB-induced genome instability in cells.

In conclusion, we show that GTE activates specific DNA repair and promotes genome stability in yeast. As such mechanisms are well-conserved between yeast and human, this study demonstrates that the yeast model may be a useful platform for future research on the underlying mechanisms of bioactive compounds in DNA repair.

## Materials and Methods

### GTE preparation

Green tea powder (purchased from a local tea company in Taiwan) (1.35 g) was mixed with warm (60 °C) distilled water (100 ml). Extraction was carried out for 20 min at 60 °C under constant stirring. The mixture was then cooled in an ice bath before being centrifuged at 4000 rpm for 5 min. The resulting supernatant was filter sterilized through a 0.22 μm Millipore^TM^ filter, yielding the GTE. Caffeine, catechin and gallic acid content were determined by HPLC using a Luna^®^ C18 reverse-phase analytical column (4.6 mm i.d. ×250 mm, 5 μm particle size; Phenomenex Inc. Torrance, CA)^48^.

### Yeast strains and growth conditions

The wild type *Saccharomyces cerevisiae* strain, BY4741 (*MATa his3Δ leu2Δ met15Δ ura3Δ*) (Euroscarf, Denmark), was used in this study. Cells were cultured in yeast extract-peptone-dextrose (YPD) media with or without EGCG (200 μM), epigallocatechin (EGC, 690 μM), caffeine (0.7 mM) or GTE, which was added at 3.5 ml GTE in 5 ml YPD culture broth. Cells were cultured for 16 h at 30 °C with shaking. Saturated cultures were used to inoculate fresh media, and the new cultures were incubated for a further 3 h at 30 °C to reach exponential phase (2 − 3 × 10^7^ cells/ml) prior to initiating experiments.

### Cell survival assay

Exponential-phase YPD cultures were diluted to an appropriate concentration, before being seeded onto YPD plates. Plates with the same cell densities, as determined by OD 600 nm, were treated with or without UVB irradiation using a UVB light source (200 or 400 J/m^2^; 365 nm peak; UVP, USA). Cells were then immediately incubated at 30 °C for 3 days in the dark. Relative survival was calculated as the ratio of colonies arising in irradiated versus control plates.

### Gene expression

Total RNA was extracted by the acid-phenol method^49^. Briefly, cells were collected and resuspended in 500 μl TES buffer (10 mM Tris-HCl pH 7.5, 10 mM EDTA, 0.5% SDS). The cell suspension was mixed with 400 μl of warm acid phenol, and incubated at 65 °C for 20 min with vortexing at 5 min intervals. Following centrifugation, the supernatant was extracted twice with acid phenol and once with chloroform. Total RNA was reverse transcribed into cDNA using random primers and the Superscript II^®^ kit (Invitrogen). Real-time PCR was performed using the ABI StepOne Plus^TM^ system. The sequences of primers used to amplify each gene are listed in Table 3. Data were normalized to *ACT1* and are presented relative to unirradiated controls. All gene expression experiments were carried out in triplicate, and two independent studies were performed.

**Table 3:** Primer sequences for gene expression and single-end invasion assay.

Gene	Primer sequences (5′ → 3′)
*RAD14*-forward	GTAAAAGGGATGCGTCGGTACT
*RAD14*-reverse	TGCATGGTGGCAAAATCGTA
*RAD4*-forward	CGATGCTCAGGGCTTGTAATG
*RAD4*-reverse	TTGGTAAAATCTGGCGGTTGA
*RAD26*-forward	GTTAAAAAAATGGGTGAAACAACGT
*RAD26*-reverse	CATTCTGGCAAGTCCGATGA
*RAD51*-forward	GCTGCCTTAGGTTCGTTTGTG
*RAD51*-reverse	CAGC AGTGTGAAGCCCACTCT
*RAD52*-forward	GCTGGTCTACGGAGGTAA
*RAD52*-reverse	ACCCTATGCTAAACTTTCCC
*RFA1*-forward	GATAACTATTTCTCAGAGCATCCAA
*RFA1*-reverse	TGGCAACATTACCACTGTC
*ACT1*-forward	TCACGCCATTTTGAGAATCG
*ACT1*-reverse	TTCAGCAGTGGTGGAGAAAGAG
*SEI*-F	TAGTCGGGTTTTTCTTTTAGTT
*SEI*-R	AAGAGGCAAGTAGATAAGGGTA

### Single-end invasion (SEI) assay

The SEI assay was performed as previously described^50^. Wild-type cells (*MATα his3Δ leu2Δ met15Δ ura3Δ*) were transformed with galactose-regulated HO nuclease expression plasmids (*p*GAL-HO; *Trp1*). Transformed cells were used to inoculate in S.C.-Trp media containing 2% raffinose, with or without GTE. Cultures were incubated at 30 °C for 24 h, to a final OD 600 of 0.6–0.8. HO endonuclease was induced by adding galactose to 2%. After 60 min, HO nuclease expression was repressed by addition of glucose to 2%. The repair intermediates were detected by real-time PCR using the extracted DNA from the cultures as templates. The fold-increase of SEI in the HO endonuclease-induced cells was calculated relative to the non-induced control. SEI assays were carried out in triplicate, and two independent studies were performed. The SEI primer sequence is shown in Table 3.

### Gene mutation assay

Gene mutation rates were determined as previously described^51^. Briefly, five to ten independent colonies were randomly selected and grown in YPD media. Cells were then plated onto either YPD to evaluate plating efficiency or synthetic complete arginine-dropout plates containing 60 mg/L canavanine. Canavanine-resistant mutants (Can^*r*^) were counted and the median mutation rates were measured^52,53^. The average fold-increase in gene mutation rate was calculated relative to wild-type cells without UV treatment as a control.

### Statistical analysis

Data are presented as the mean ± S.D. Comparisons were performed using Student’s *t*-test. Gene mutation experiments were evaluated using the Mann-Whitney method. Statistical significance was set as *P* < 0.05.

## Data Availability

The datasets generated during the current study are available from the corresponding author on reasonable request.
